# Dietary Exposure and Risk Assessment for L-Ergothioneine in China

**DOI:** 10.3390/foods15050822

**Published:** 2026-03-01

**Authors:** Sheng Ma, Xiaochen Ma, Ling Hao, Ling Yong, Tong Ou, Xiao Xiao, Bingwen Yi, Weichunbai Zhang, Yan Song

**Affiliations:** 1 NHC Key Laboratory of Food Safety Risk Assessment, China National Center for Food Safety Risk Assessment, Beijing 100022, China; masheng@pku.org.cn (S.M.); yongling@cfsa.net.cn (L.Y.); outong@cfsa.net.cn (T.O.); xiaoxiao@cfsa.net.cn (X.X.); bingwen8589@163.com (B.Y.); 2 Beijing Center for Disease Control and Prevention, Institute of Nutrition and Food Hygiene, Beijing 100013, China; xiaoch-ma@126.com (X.M.); hl_aoing@163.com (L.H.); 3 School of Public Health, Southern Medical University, Guangzhou 510515, China

**Keywords:** L-ergothioneine, dietary exposure, risk assessment, edible fungi, margin of exposure, Chinese population

## Abstract

L-Ergothioneine (L-EGT), a naturally occurring thiol compound abundant mainly in edible fungi, is increasingly regarded as a potentially beneficial bioactive constituent. However, population-level exposure data remain limited. This study aimed to estimate background dietary exposure to L-EGT among Chinese residents, describe its distribution across population subgroups and regions, identify major food contributors, and characterize the risk using a margin of exposure (MOE) approach. Individual body-weight-normalized L-EGT intakes were estimated from published food concentration data and three-day dietary recalls of 42,218 participants. MOEs were calculated using a no observed adverse effect level (NOAEL) of 800 mg/kg bw/d obtained from subchronic toxicity studies. The mean dietary exposure to L-EGT was 0.043 mg/kg bw/d (MOE = 18,605) in the general population and 0.174 mg/kg bw/d (MOE = 4598) among consumers, with 95th percentile exposures of 0.244 mg/kg bw/d (MOE = 3279) and 0.644 mg/kg bw/d (MOE = 1242), respectively. MOE values were consistently above the safety threshold of 300 across all subgroups, with less than 0.3% of the total population and 1.3% of consumers aged 3–6 years falling below this value. These results indicate that current natural dietary exposure to L-EGT in China is low and does not raise safety concerns and provide essential baseline data for future studies on its health effects, optimal intake ranges, and long-term safety.

## 1. Introduction

L-Ergothioneine (L-EGT) (C_9_H_15_N_3_O_2_S) is a naturally occurring, sulfur-containing derivative of histidine that was first isolated from ergot fungus by French researchers in 1909 [1]. Because humans lack the capacity for its endogenous synthesis, L-EGT must be obtained solely from the diet, where it is ubiquitously distributed in sources such as edible mushrooms, certain legumes, and animal offal [2]. However, it is important to note that L-EGT is not currently classified as an essential nutrient, as no specific deficiency syndrome has been definitively established. Upon ingestion, L-EGT is actively transported into tissues via the specific transporter OCTN1 and preferentially accumulates in tissues and cellular compartments subjected to elevated oxidative or inflammatory stress, including the bone marrow, liver, kidneys, brain, and ocular lens [1,3,4]. This selective distribution, coupled with its extended half-life of approximately one month, suggests a conserved physiological function in the regulation of redox homeostasis and cytoprotection [1,2].

Over the past decade, L-EGT has attracted increasing attention due to its broad antioxidant, anti-inflammatory, and cytoprotective properties and observational data linking additional L-EGT intake or plasma L-EGT levels with lower risks of cognitive decline, sleep disorders, and cardiometabolic diseases [1,5,6]. In vitro experimental studies indicate that L-EGT can scavenge reactive oxygen species, mitigate oxidative DNA damage, and modulate endothelial function [7,8,9,10,11,12], while animal models and human supplementation trials show effects on biomarkers of oxidative stress and inflammation [4,13,14,15,16]. These converging lines of evidence have encouraged researchers to explore strategies for assessing and enhancing L-EGT intake, such as incorporation into food products, to harness its potential health benefits in humans.

Alongside this growing scientific interest, regulatory scrutiny has also focused on L-EGT. There is currently no internationally established health-based guidance value (HBGV) for L-EGT. The European Food Safety Authority (EFSA) affirmed the safety of synthetic L-EGT as a novel food based on a no observed adverse effect level (NOAEL) of 800 mg/kg body weight (bw)/day from subchronic studies, yielding margins of exposure (MOE) well above 200 even for high consumers [17]. The approved use of L-EGT by the European Union (EU) allows for up to 5 mg per serving in various products, including alcohol-free beverages, cereal bars, milk, fresh dairy products, and chocolate. The proposal also includes its use as a food supplement, with a maximum daily dose of 30 mg for adults and 20 mg for children [18,19]. In the United States, the Food and Drug Administration (FDA) classifies L-EGT as a Generally Recognized As Safe (GRAS) substance and permits its use in products such as cakes, cookies, and so on [20,21]. In addition, L-EGT is listed in the “Non-Medicinal Ingredients” catalog in Japan, allowing its use in foods on the condition that no health claims are made, with no specific limits set on its use [22]. Canada has also approved L-EGT as a natural health product ingredient for preservation and antioxidant purposes, with intake limits of 6 mg per day for individuals aged 14 and above and 2.2 mg for children aged 1–13 years, without claims of antioxidant effects [23]. In China, L-EGT has been submitted for approval as a Novel Food Ingredient [24]. These opinions emphasize the need for reliable information on natural dietary exposure to L-EGT in different populations to inform both risk assessment and benefit–risk appraisal of fortified foods and supplements.

Despite growing interest in L-EGT as a bioactive dietary component, quantitative data on background exposure at the population level remain limited and are largely derived from European or North American food composition datasets and dietary patterns [25]. In particular, little is known about habitual L-EGT intake in Asian populations, where culinary traditions include high levels of consumption of edible fungi, soy products, legumes, and organ meats—food groups that may collectively contribute to comparatively higher L-EGT exposure. However, no study has systematically compiled L-EGT concentrations in foods commonly consumed in China and linked these data to nationally representative dietary intake surveys to characterize comprehensive background exposure and its variability across sociodemographic subgroups.

Therefore, this study aimed to compile published data on L-EGT concentrations in foods relevant to the Chinese diet, integrate these with nationally representative dietary consumption data, and estimate dietary exposure to L-EGT among the Chinese population. A subsequent risk characterization was also conducted using available toxicological reference values. These findings are intended to provide a scientific basis for future evaluations of L-EGT as a potentially beneficial bioactive constituent in the Chinese food supply and to support risk assessment and regulatory decision making related to its use in novel foods and ingredients.

## 2. Materials and Methods

### 2.1. Database of L-EGT Concentrations in Foods

A database of L-EGT concentrations in foods was compiled from the published literature following a pre-specified hierarchical selection strategy. For edible fungi, the predominant dietary source of L-EGT, Chinese studies were prioritized based on more advanced detection techniques and more recent publication dates. The study by E et al. (2022) [26], employing Ultra Performance Liquid Chromatography–Hydrophilic Interaction Chromatography (HILIC-UPLC) (Milford, MA, USA), served as the primary reference. Fungal species not covered in that study were supplemented with data from Zhang et al. (2013) [27]. For non-fungal food categories lacking Chinese analytical data, the study by Cheah et al. (2018) [28], conducted in Singapore with an East Asian dietary context most comparable to that of China, was used as the main source. Data for remaining items were supplemented by the study by Ey et al. (2007) [29]. All major food categories with reported detections were included: fungi, vegetables, dried beans and products, animal offal, and nuts and seeds. Foods with concentrations below 1 mg/kg wet weight or non-detectable levels were excluded, as such foods contribute negligibly to total L-EGT exposure, consistent with the EFSA approach [17] of restricting background exposure assessment primarily to edible fungi.

For foods lacking moisture content in source publications, values were imputed from the Chinese Food Composition Table (6th edition, 2023) [30] for corresponding items. If unavailable therein, data from the U.S. Department of Agriculture FoodData Central (available online: https://fdc.nal.usda.gov/, accessed on 5 September 2025) or NutriData database (available online: https://www.nutridata.cn/home, accessed on 5 September 2025) were used as proxies. Converted concentrations were applied uniformly to align with the consumption data. Wet-to-dry weight conversions were standardized using the following formula:

(1) $$C_{wet}=C_{dry}\times \frac{\left(100-M\right)}{100}$$

where $C_{wet}$ is the concentration on a wet weight basis (mg/kg), $C_{dry}$ is the dry weight concentration (mg/kg), and M is the moisture content (%).

### 2.2. Food Consumption and Body Weight Data

Food consumption data were obtained from the 2018–2020 Chinese Residents Food Consumption Survey [31], a pre-existing dataset collected by the China National Center for Food Safety Risk Assessment. The nationally representative study employed a multistage random sampling method and covered 48 investigation points in 21 provinces, autonomous regions, or municipalities. The target population consisted of usual household members aged ≥3 years. Individuals with major diseases that had substantially altered their usual diet, or who were unable to complete the dietary interview or anthropometric measurements, were excluded. Basic demographic information collection and non-consecutive 3-day, 24-h dietary recalls were conducted by trained interviewers. For each of the 3 days, all foods and beverages consumed by respondents in the preceding 24 h were recorded, encompassing both home-prepared and out-of-home items. Reported intakes were converted into gram weights of individual food items using standardized recipes and portion sizes. For each individual, the daily intake of each food was calculated as the mean across the three survey days.

Body weight was measured on site by trained staff using portable electronic scales with a precision of 0.1 kg, following standardized procedures. When on-site measurement was not feasible, self-reported recent body weight was used. After exclusion of implausible body weight values (n = 20), a total of 42,218 participants were included in the analysis.

### 2.3. Dietary Exposure Assessment

Dietary exposure to L-EGT from natural foods was estimated using a simple distributional approach to derive individual-level chronic intakes normalized to body weight. For each participant, exposure was calculated as

(2) $$Exp=\sum_{i=1}^{n}\frac{\left(F_{i}\times C_{i}\right)}{W}$$

where Exp is the individual daily L-EGT intake (mg/kg bw/d); $F_{i}$ denotes the consumption of food items by an individual (kg/day); $C_{i}$ is the mean L-EGT concentration in food (mg/kg); and W is the individual body weight (kg).

### 2.4. Risk Characterization Approach

Given the absence of a HBGV for L-EGT, the margin of exposure (MOE) was calculated using an NOAEL of 800 mg/kg bw/d derived from subchronic toxicological studies, consistent with EFSA evaluations. The MOE for each individual was defined as

(3) $$\mathrm{MOE}=\frac{\mathrm{NOAEL}}{\mathrm{Exp}}$$

where NOAEL is 800 mg/kg bw/d and Exp is the estimated individual dietary exposure as defined above. Based on the recommendation of the EFSA Scientific Committee, a MOE cut-off value of 300 was applied for the risk characterization of L-EGT, which is composed of a 10-fold factor for inter-species variation, a 10-fold factor for human inter-individual variation, and a 3-fold factor for temporal extrapolation [32].

### 2.5. Statistical Analysis

Dietary exposure to L-EGT and MOEs were calculated for the general population (all participants) and the consuming population (participants who consumed at least one L-EGT-containing food during the 3-day recall). Distributions were summarized overall and stratified by sex, age group, and province, with mean and 95th percentile (*P*_95_) values of Exp (mg/kg bw/d) and MOE reported. The proportion of individuals with MOEs below 300 was also calculated. All analyses were performed in R (version 4.4.2).

## 3. Results

### 3.1. L-EGT Concentrations in Food

**Figure 1:**
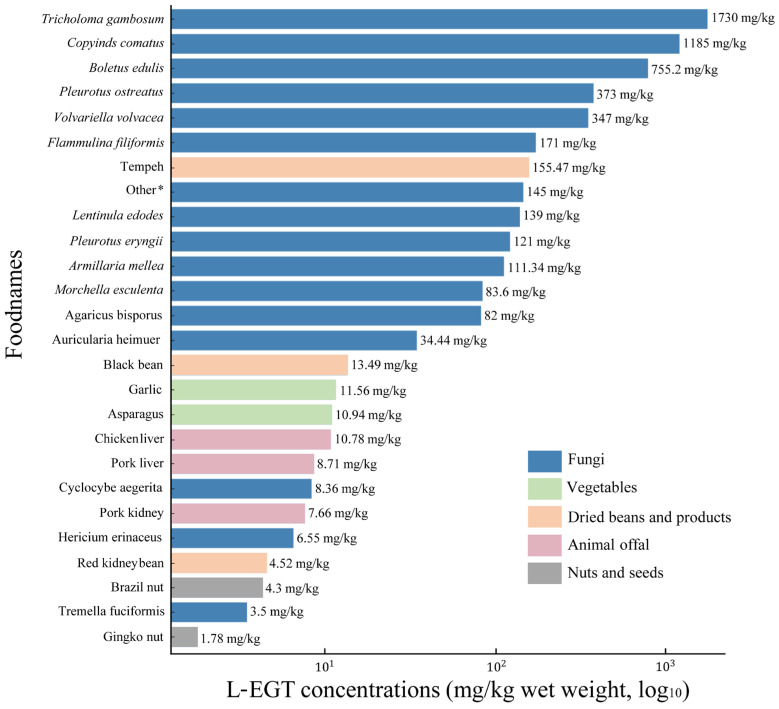
L-EGT concentrations in different foods (mg/kg wet weight) [26,27,28,29]. Note: * represents the mean concentrations of white *H. marmoreus*, *P. cornucopiae*, *H. marmoreus*, and *S. rugoso-annulata*.

L-EGT concentrations in different food groups are shown in Figure 1 and Table S1. Fungi exhibited by far the highest levels. Among fresh mushrooms, *Tricholoma gambosum* and *Coprinus comatus* showed the greatest L-EGT concentrations on a wet-weight basis (1730 and 1185 mg/kg, respectively), followed by *Boletus edulis* (775.2 mg/kg). Commonly consumed cultivated mushrooms, such as *Pleurotus ostreatus* (373 mg/kg), *Volvariella volvacea* (347 mg/kg), and *Flammulina filiformis* (171 mg/kg), also contained substantial L-EGT levels. In contrast, species like *Cyclocybe aegerita*, *Hericium erinaceus,* and *Tremella fuciformis* had lower concentrations (3.5–8.36 mg/kg wet weight).

Non-fungal foods generally contained much lower L-EGT levels. Among vegetables, garlic and asparagus contained about 11.6 and 10.9 mg/kg wet weight, respectively. In dried beans and products, tempeh had a relatively high concentration (155.47 mg/kg), while black beans and red kidney beans contained 13.49 and 4.52 mg/kg, respectively. Animal offal, such as chicken and pork liver and kidney, contained L-EGT in the range of 7.66–10.78 mg/kg. Nuts and seeds had the lowest levels, with Brazil nuts and ginkgo nuts containing 4.30 and 1.78 mg/kg, respectively.

### 3.2. Food Consumption

**Figure 2:**
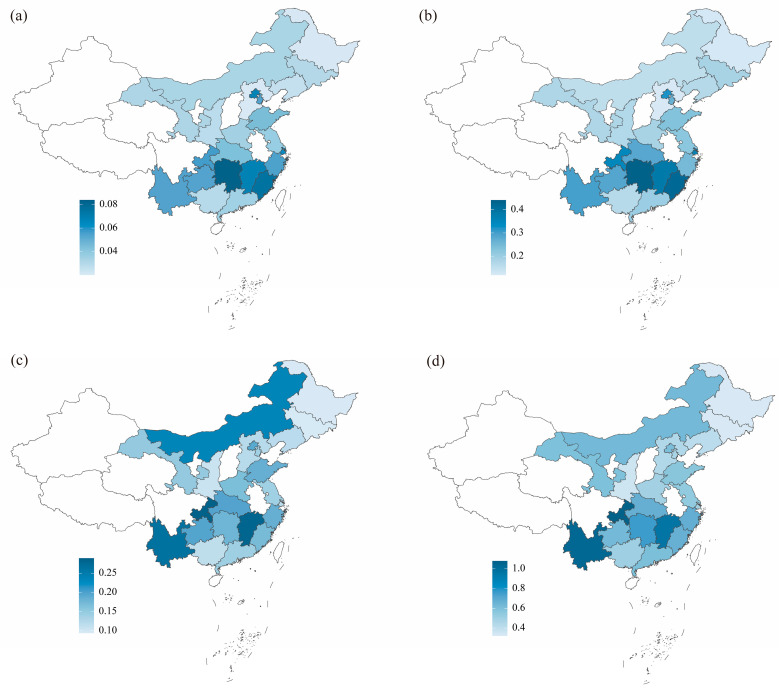
Dietary intake levels of L-EGT in different regions (mg/kg bw/d). (**a**) Mean intake in the general population; (**b**) 95th percentile (*P*_95_) intake in the general population; (**c**) mean intake in the consuming population; (**d**) *P*_95_ intake in the consuming population. The color gradient from light to dark blue indicates increasing intake levels. Regions shown in white represent provinces, autonomous regions, or municipalities for which data were not available. The base map was obtained from the National Platform for Common GeoSpatial Information Services (https://cloudcenter.tianditu.gov.cn/administrativeDivision, accessed on 8 December 2025; Drawing Approval Number: GS (2024) 0650).

Daily consumption of L-EGT-containing foods among the general and consuming population is summarized in Table 1. The bubble plot in Figure S1 contrasts mean L-EGT concentrations with consumption volumes. Among fungi, *Lentinula edodes*, *Flammulina filiformis*, and *Armillaria mellea* showed the highest intake in the general population and substantially higher levels among consumers. Most other fungi, dried products, beans, vegetables, nuts, and animal offal exhibited low mean intakes, generally below 1 g/d. Pork liver was an exception, with 1.56 g/d in the general population and a *P*_95_ of 50 g/d among consumers. As shown in Figure 2, several edible fungi exhibited both high L-EGT concentrations and comparatively higher daily consumption.

### 3.3. Dietary Exposure to L-EGT

Dietary intake levels of L-EGT in the Chinese population by sex and age group are shown in Table 2. For the general population (n = 42,218), mean exposure was 0.043 mg/kg bw/d, with a *P*_95_ of 0.244 mg/kg bw/d. Meanwhile, among the consuming population (n = 10,518), mean exposure increased to 0.174 mg/kg bw/d and *P*_95_ to 0.644 mg/kg bw/d. Females consistently had slightly higher L-EGT exposure than males. In the general population, female mean intake was 0.047 mg/kg bw/d (*P*_95_ = 0.264 mg/kg bw/d) compared with 0.039 mg/kg bw/d (*P*_95_ = 0.222 mg/kg bw/d) in males. Age-stratified analyses revealed that children had the highest body-weight-adjusted L-EGT exposure. Among 3–6-year-olds, mean intake in the general population was 0.094 mg/kg bw/d (*P*_95_ = 0.522 mg/kg bw/d), and in the consuming population it was 0.364 mg/kg bw/d (*P*_95_ = 1.306 mg/kg bw/d). For 7–12-year-olds, mean exposure was 0.061 mg/kg bw/d (*P*_95_ = 0.356 mg/kg bw/d) in the general population and 0.249 mg/kg bw/d (*P*_95_ = 0.869 mg/kg bw/d) in consumers. Adolescents and middle-aged adults (13–44 years) exhibited intermediate values, whereas adults (≥45 years) had lower mean exposures of approximately 0.031–0.036 mg/kg bw/d and consuming population means around 0.131–0.160 mg/kg bw/d.

### 3.4. Regional Variation in L-EGT Exposure

Substantial inter-provincial variation in dietary L-EGT exposure was observed (Figure 2, Table S2). In the general population, mean exposures ranged from about 0.020 mg/kg bw/d in provinces such as Hebei and Heilongjiang to 0.076–0.084 mg/kg bw/d in Fujian and Hunan, with several coastal and central provinces (e.g., Beijing, Shanghai, Jiangxi, Yunnan, Chongqing) showing intermediate to high values (0.054–0.068 mg/kg bw/d). When focusing on the consuming population, mean exposures exceeded 0.26 mg/kg bw/d in Yunnan and Chongqing and approached 0.28–0.29 mg/kg bw/d in Jiangxi and Chongqing, with P95 values around or above 1.0 mg/kg bw/d in provinces such as Yunnan and Chongqing.

### 3.5. Major Food Contributors to L-EGT Exposure

Decomposition of exposure by food group confirmed that fungi overwhelmingly dominated L-EGT intake (Table 3). In the general population, fungi accounted for 97.7% of total L-EGT exposure on average and 98.4% at the *P*_95_. In the consuming population, the contribution of fungi remained above 97% for the mean and almost 99.5% at the *P*_95_. At the species level, *Lentinula edodes* was the single largest source of L-EGT, contributing 34.9% of mean and 28.7% of *P*_95_ exposure in the general population and 34.5% and 44.1% of mean and *P*_95_ exposure in the consuming population. *Auricularia heimuer* contributed 18.6% (mean) and 13.1% (*P*_95_) of exposure in the general population and 18.4% and 22.5% in consumers. *Pleurotus ostreatus* and *Flammulina filiformis* each contributed approximately 13–14% of mean exposure, with *Flammulina filiformis* becoming particularly important at the high end of intake in the consuming population (27.5% at *P*_95_). Other mushroom species together accounted for about 7% of intake. In contrast, vegetables, dried beans and products, animal offal, and nuts and seeds each contributed ≤ 2–3% of total L-EGT exposure on average.

### 3.6. Risk Characterization

MOE values for L-EGT across different population groups are presented in Table 4. In the general population, the overall mean MOE was 18,605 and the *P*_95_ MOE was 3279. Sex-specific mean MOEs were 20,513 for males and 17,021 for females, with *P*_95_ values being 3604 and 3030, respectively, and only 0.1% of individuals had MOEs below 300. As for age groups, children and adolescents (3–17 years old) had lower MOE values, but, even with *P*_95_ intake, the MOE value is still greater than 1500. Adult age groups (18–44, 45–59, ≥60 years) had higher MOEs, with mean values typically between 16,000 and 25,806 and *P*_95_ values close to or above 3300–4100.

In the consuming population, the overall mean MOE was 4598, with a *P*_95_ of 1242. Sex-specific *P*_95_ MOEs were 1342 for males and 1187 for females, with 0.2–0.4% of individuals below 300. Children aged 3–6 years showed the lowest MOEs, reflecting their highest exposure per kg body weight. In the consuming population, mean and *P*_95_ MOEs were 2198 and 613, respectively, with 1.3% of individuals below 300. For 7–12-year-olds, the corresponding values were 3213 (mean) and 921 (*P*_95_), and 0.2% were below 300. In other age–sex strata, the proportion of individuals with MOE < 300 was 0–0.5%.

## 4. Discussion

The present study provides the first comprehensive, population-based assessment of dietary L-EGT exposure among Chinese residents, combining compiled concentration data with nationally representative dietary consumption patterns. Overall exposure from natural foods was low, with mean intakes of 0.043 mg/kg bw/d in the general population and 0.174 mg/kg bw/d among consumers. Edible fungi accounted for more than 97% of total L-EGT intake. MOE analysis revealed a wide safety margin. The mean MOE value for the general population (18,605) far exceeded the safety threshold of 300, and even at the 95th percentile among 3–6-year-old consumers, the group with the highest body-weight-adjusted intakes, the MOE remained above 600, indicating negligible health risks under current dietary conditions.

Direct comparison with other countries is challenging due to the scarcity of exposure assessments and heterogeneity in food composition data. Nonetheless, existing estimations from Europe and the United States suggest mean daily L-EGT intakes from the background diet ranging from 0.016 to 0.067 mg/kg bw/d in the general population and 0.057 to 0.244 mg/kg bw/d among consumers. At the 95th percentile, exposure among adult consumers was reported to be 0.203–0.660 mg/kg bw/d, with child consumers being 1.017–1.110 mg/kg bw/d [25]. Derived primarily from mushroom consumption data, the 2016 EFSA scientific opinion reported that Italy had the highest estimated exposures in Europe, with adults’ *P*_95_ values up to 0.48–0.70 mg/kg bw/day and child *P*_95_ levels up to 0.64–1.11 mg/kg bw/d [17]. Despite differences in dietary patterns, the preliminary comparison tentatively suggests that natural dietary L-EGT intake may be broadly similar between Chinese and Western populations.

The high MOE values observed indicate a wide margin between current dietary exposure and the NOAEL of 800 mg/kg bw/d. Children exhibited the highest body-weight-adjusted exposure, with *P*_95_ intakes reaching 0.522 mg/kg bw/d (3–6 years) and 0.356 mg/kg bw/d (7–12 years). This age-dependent pattern aligns with established observations for other nutrients and contaminants, resulting from smaller body mass and diverse mushroom-containing diets. Nevertheless, even among young children, MOE values far exceeded 300, indicating a low health risk. Regional variability in L-EGT exposure was pronounced. Higher exposure levels in provinces such as Yunnan, Jiangxi, Fujian, and Chongqing reflect both dietary traditions rich in edible fungi and local availability of wild or dried mushrooms. Conversely, northern regions such as Hebei and Heilongjiang showed low exposures consistent with their culinary patterns.

It should be noted that the wide safety margins demonstrated above pertain to toxicological risk, not physiological efficacy. These are distinct considerations: the absence of safety concern at background exposure levels does not imply that such intakes are sufficient to confer measurable health benefits. Existing human studies suggest that daily intakes below 25 mg are well-tolerated and may be associated with improvements in cognitive performance, sleep quality, and joint-related outcomes [4,16,33,34,35,36,37,38,39]. However, the limited number of trials and heterogeneous endpoints do not allow for a robust dose–response meta-analysis to define an efficacy threshold of L-EGT intake. In one randomized controlled trial supported by a physiologically based pharmacokinetic model [36], a regimen of 8 mg/day for 16 weeks was identified as an optimal dose to achieve putatively effective plasma L-EGT concentrations to improve subjective sleep outcomes, implying that background intakes from unfortified foods are unlikely to reach levels associated with physiological benefits in most individuals. To date, several regulatory authorities, including the EU, the United States, Japan, and Canada, have already authorized the addition of L-EGT to foods, dietary supplements, or natural health products [18,19,20,21,22,23,40]. Regarding safety, the EFSA has evaluated high consumption levels of L-EGT from fortified foods, background diet, and food supplements. For adults, estimated high intakes are 0.545, 0.700, and 0.430 mg/kg bw, respectively, resulting in a combined total of 1.68 mg/kg bw (corresponding MOE = 470). For children, the values are 1.179, 1.110, and 1.429 mg/kg bw, respectively, with a combined total of 3.72 mg/kg bw (corresponding MOE = 216). The EFSA concluded that the resulting margins of safety are sufficient [17]. Our study suggests that the existing natural dietary exposure in China is unlikely to pose health risks while at the same time providing a quantitative basis for risk characterization, future novel food applications, and benefit–risk assessment of L-EGT-containing foods. Establishing such population-level baselines is also essential for interpreting potential health benefits in epidemiologic and clinical studies.

There are some uncertainties in this study. First, regarding food concentration data, uncertainties arise from heterogeneity across source studies in sampling years and areas, extraction and chromatographic methods, and limits of quantification, all of which may introduce between-study measurement variability. Additional uncertainty is introduced when converting between dry-weight and wet-weight concentrations using standard water contents from food composition tables, especially for foods with variable moisture. Moreover, potential changes in L-EGT content during processing, cooking, soaking, or rehydration were not comprehensively quantified. Secondly, for consumption data, the 3-day, 24-h recall design is subject to recall bias, and self-reported body weight, where used, may deviate from measured values and hence affect mg/kg bw exposure estimates. Finally, non-dietary exposure routes, such as cosmetic use, could, in principle, contribute to limited local skin exposure.

Future work should build on these baseline exposure estimates in a stepwise manner. First, China-specific L-EGT concentration data should be expanded beyond edible fungi through systematic sampling across regions and seasons and by quantifying the effects of processing and cooking to reduce uncertainty in intake estimation. Second, the population-level baseline established here provides a foundation for prospective cohort studies examining associations between L-EGT intake and health outcomes, including cognitive decline, cardiometabolic disease, and healthy aging. Special attention should be given to potentially vulnerable or high-benefit subgroups, such as pregnant women, older adults, and individuals with chronic diseases. Third, the development and harmonization of standardized analytical methods, including, in particular, optimized LC-MS/MS protocols for food matrices and biological samples, will be critical to ensure cross-study comparability. Finally, as regulatory interest in L-EGT as a novel food ingredient grows in China, combined exposure estimates incorporating fortified foods and supplements will be essential for benefit–risk assessment and evidence-based regulatory decision making.

## 5. Conclusions

This nationwide assessment provides the first comprehensive characterization of background dietary exposure to L-EGT among Chinese residents. Chronic exposure to L-EGT from natural foods was low, and MOE analysis indicated a wide safety margin across all demographic strata, even among young children who exhibited the highest body-weight-adjusted intakes. Edible fungi contributed more than 97% of total L-EGT exposure, with commonly consumed mushrooms—*Lentinula edodes*, *Auricularia heimuer*, and *Pleurotus ostreatus*—as the main dietary sources. These findings indicate that current background dietary exposure to L-EGT in China does not raise safety concerns and provide a quantitative basis for risk characterization, future novel food applications, and benefit–risk assessment of L-EGT-containing foods in Chinese regulatory and public health settings.

## Figures and Tables

**Table 1:** Consumption of different foods among the general and consuming population (g/d).

Food Groups	No. of Consumers	General Population (n = 42,218)		Consuming Population (n = 10,518)	
		Mean	*P*_95_	Mean	*P*_95_
Fungi					
*Tricholoma gambosum*	20	0.03	0	0.13	0
*Copyinds comatus*	60	0.10	0	0.40	0
*Boletus edulis*	32	0.05	0	0.20	0
*Pleurotus ostreatus*	506	0.82	0	3.27	0
*Volvariella volvacea*	46	0.07	0	0.29	0
*Flammulina filiformis*	1226	1.76	0	7.08	52.00
*Lentinula edodes*	2371	2.94	10.00	11.81	80.00
*Lentinula edodes* (dry)	544	0.21	0	0.84	2.00
*Pleurotus eryngii*	375	0.66	0	2.65	0
*Armillaria mellea*	14	0.01	0	0.05	0
*Armillaria mellea* (dry)	1	0.00	0	0.01	0
*Morchella esculenta* (dry)	27	0.03	0	0.13	0
*Agaricus bisporus*	115	0.14	0	0.57	0
*Auricularia heimuer*	1726	1.65	0	6.64	50.00
*Auricularia heimuer* (dry)	2726	0.83	3.00	3.32	20.00
*Cyclocybe aegerita* (dry)	120	0.08	0	0.30	0
*Hericium erinaceus*	16	0.01	0	0.05	0
*Tremella fuciformis* (dry)	320	0.16	0	0.63	0
Other	144	0.23	0	0.93	0
Vegetables					
Garlic	331	0.23	0	0.94	0
Garlic (dry)	12	0.01	0	0.05	0
Asparagus	220	0.44	0	1.76	0
Dried beans and products					
Tempeh	402	0.21	0	0.85	0
Black bean	151	0.09	0	0.36	0
Red kidney bean	88	0.13	0	0.51	0
Animal offal					
Chicken liver	44	0.07	0	0.26	0
Pork liver	953	1.56	0	6.27	50.00
Pork kidney	158	0.27	0	1.09	0
Nuts and seeds					
Brazil nut	41	0.06	0	0.23	0
Gingko nut	23	0.02	0	0.09	0

Note: Brazil nut consumption was replaced by consumption of other nuts.

**Table 2:** Dietary intake levels of L-EGT in different populations (mg/kg bw/d).

Groups	General Population			Consuming Population		
	n	Mean	*P*_95_	n	Mean	*P*_95_
Total	42,218	0.043	0.244	10,518	0.174	0.644
Males	20,423	0.039	0.222	5050	0.159	0.596
Females	21,795	0.047	0.264	5468	0.187	0.674
3–6 years	2583	0.094	0.522	670	0.364	1.306
7–12 years	3573	0.061	0.356	871	0.249	0.869
13–17 years	1722	0.043	0.242	396	0.187	0.792
Boys	881	0.041	0.232	218	0.166	0.672
Girls	841	0.045	0.271	178	0.213	0.902
18–44 years	14,324	0.043	0.238	3882	0.159	0.565
Males	6601	0.035	0.194	1748	0.133	0.465
Females	7723	0.050	0.275	2134	0.181	0.645
45–59 years	13,312	0.032	0.202	3154	0.137	0.514
Males	6433	0.031	0.190	1508	0.131	0.472
Females	6879	0.034	0.211	1646	0.142	0.546
≥60 years	6704	0.036	0.199	1545	0.156	0.552
Males	3287	0.036	0.199	783	0.152	0.517
Females	3417	0.036	0.198	762	0.160	0.569

**Table 3:** L-EGT exposure from different food groups (mg/kg bw/d).

Food Groups	General Population (n = 42,218)				Consuming Population (n = 10,518)			
	Mean	Percentage/%	*P*_95_	Percentage/%	Mean	Percentage/%	*P*_95_	Percentage/%
Total	0.043	100.0	0.244	100.0	0.174	100.0	0.644	100.0
Fungi	0.042	97.7	0.240	98.4	0.169	97.1	0.641	99.5
*Lentinula edodes*	0.015	34.9	0.070	28.7	0.060	34.5	0.284	44.1
*Auricularia heimuer*	0.008	18.6	0.032	13.1	0.032	18.4	0.145	22.5
*Pleurotus ostreatus*	0.006	14.0	0	0	0.024	13.8	0.000	0.0
*Flammulina filiformis*	0.006	14.0	0	0	0.023	13.2	0.177	27.5
*Copyinds comatus*	0.002	4.7	0	0	0.009	5.2	0	0
*Pleurotus eryngii*	0.001	2.3	0	0	0.006	3.4	0	0
*Tricholoma gambosum*	0.001	2.3	0	0	0.004	2.3	0	0
All other	0.003	7.0	-	-	0.011	6.3	-	-
Vegetables	0	0.0	0	0	0.001	0.6	0.001	0.2
Dried beans and products	0.001	2.3	0	0	0.003	1.7	0.002	0.3
Animal offal	0	0.0	0	0	0.001	0.6	0.009	1.4
Nuts and seeds	0	0.0	0	0	0	0	0	0

**Table 4:** Margin of exposure (MOE) for L-EGT across different population groups.

Groups	General Population (n = 42,218)			Consuming Population (n = 10,518)		
	Mean	*P*_95_	MOE < 300 (%)	Mean	*P*_95_	MOE < 300 (%)
Total	18,605	3279	0.1	4598	1242	0.3
Males	20,513	3604	0.1	5031	1342	0.2
Females	17,021	3030	0.1	4278	1187	0.4
3–6 years	8511	1533	0.3	2198	613	1.3
7–12 years	13,115	2247	0.1	3213	921	0.2
13–17 years	18,605	3306	0.0	4278	1010	0.0
Boys	19,512	3448	0.0	4819	1190	0.0
Girls	17,778	2952	0.0	3756	887	0.0
18–44 years	18,605	3361	0.1	5031	1416	0.3
Males	22,857	4124	0.0	6015	1720	0.1
Females	16,000	2909	0.1	4420	1240	0.4
45–59 years	25,000	3960	0.0	5839	1556	0.0
Males	25,806	4211	0.0	6107	1695	0.1
Females	23,529	3791	0.0	5634	1465	0.0
≥60 years	22,222	4020	0.1	5128	1449	0.5
Males	22,222	4020	0.1	5263	1547	0.5
Females	22,222	4040	0.1	5000	1406	0.4

## Data Availability

The raw data supporting the conclusions of this article will be made available by the authors upon request.

## Supplementary Materials

The following supporting information can be downloaded at https://www.mdpi.com/article/10.3390/foods15050822/s1, Table S1: L-EGT concentrations in foods (mg/kg); Figure S1: Bubble plot of the mean concentration of L-EGT in foods versus their consumption; Table S2: Dietary intake levels of L-EGT in different regions (mg/kg bw/d).

## Abbreviations

The following abbreviations are used in this manuscript:

L-EGT	L-Ergothioneine
MOE	Margin of Exposure
NOAEL	No Observed Adverse Effect Level
FDA	Food and Drug Administration
GRAS	Generally Recognized as Safe
UPLC	Ultra-Performance Liquid Chromatography
HILIC	Hydrophilic Interaction Liquid Chromatography
LC-MS	Liquid Chromatography–Mass Spectrometry
LC-MS/MS	Liquid Chromatography–Tandem Mass Spectrometry
