# Supplementary material for: Dietary Exposure and Risk Assessment for L-Ergothioneine in China
Source: Foods. 2026 Mar 1;15(5):822. doi: 10.3390/foods15050822 (PMC12985124; doi:10.3390/foods15050822)
Supplement: Supplementary file 1 [file foods-15-00822-s001.zip › Table S1.pdf]

Table S1. L-EGT concentrations in foods (mg/kg).

Latin names	Chinese names	Dry content (mg/kg)	water content (%)	Fresh content (mg/kg)	detection method	Reference
Fungi	-					
Tricholoma gambosum	Kou'mo	22763	92.4	1730^{+}	LC-MS	Zhang et al. 2013[27]
Copyinds comatus	Ji'tui'gu	15592	92.4	1185^{+}	LC-MS	Zhang et al. 2013[27]
Boletus edulis	Niu'gan'jun	10200^{+}	92.4	775.2	HILIC-UPLC	E et al. 2022[26]
Pleurotus ostreatus	Ping'gu	4973	92.5	373^{+}	HILIC-UPLC	E et al. 2022[26]
Volvariella volvacea	Cao'gu	4566	92.4	347^{+}	HILIC-UPLC	E et al. 2022[26]
Flammulina filiformis	Jin'zhen'gu	1745	90.2	171^{+}	HILIC-UPLC	E et al. 2022[26]
Lentinula edodes	Xiang'gu	1675	91.7	139^{+}	HILIC-UPLC	E et al. 2022[26]
Pleurotus eryngii	Xing'bao'gu	1163	89.6	121^{+}	HILIC-UPLC	E et al. 2022[26]
Armillaria mellea	Zhen'mo	1465^{+}	92.4	111.34	HILIC-UPLC	E et al. 2022[26]
Morchella esculenta	Yang'du'jun	1100^{+}	92.4	83.6	HILIC-UPLC	E et al. 2022[26]
Agaricus bisporus	Shuang'bao'gu	1079	92.4	82^{+}	HILIC-UPLC	E et al. 2022[26]
Auricularia heimuer	Hei'mu'er	420^{+}	91.8	34.44	HILIC-UPLC	E et al. 2022[26]
Cyclocybe aegerita	Cha'shu'gu	110^{+}	92.4	8.36	HILIC-UPLC	E et al. 2022[26]
Hericium erinaceus	Hou'tou'gu	85^{+}	92.3	6.55	HILIC-UPLC	E et al. 2022[26]
Tremella fuciformis	Yin'er	46^{+}	92.4	3.5	HILIC-UPLC	E et al. 2022[26]
Other*	-	1908	92.4	145^{+}	HILIC-UPLC	E et al. 2022[26]
Vegetables	-					
Garlic	-	34.6^{+}	66.6	11.56	LC-MS	Barry et al. 2018[28]
Asparagus	-	163.25^{+}	93.3	10.94	LC-MS	Barry et al. 2018[28]
Dried beans and products	-					
Tempeh	-	201.13^{+}	22.7	155.47	LC-MS	Barry et al. 2018[28]
Black bean	-	15.02	10.2	13.49^{+}	LC-MS/MS	Ey et al. 2007[29]
Red kidney bean	-	-	-	4.52^{+}	LC-MS/MS	Ey et al. 2007[29]
Nuts and seeds	-					
Brazil nut	-	4.45^{+}	3.42	4.30	LC-MS	Barry et al. 2018[28]
Gingko nut	-	3.98^{+}	55.2	1.78	LC-MS	Barry et al. 2018[28]
Animal offal	-					
Chicken liver	-	-	-	10.78^{+}	LC-MS/MS	Ey et al. 2007[29]
Pork liver	-	-	-	8.71^{+}	LC-MS/MS	Ey et al. 2007[29]
Pork kidney	-	-	-	7.66^{+}	LC-MS/MS	Ey et al. 2007[29]

Note: *represents the mean concentrations of white *H.marmoreus*, *P.cornucopiae, H.marmoreus*, and *S.rugoso-annulata*;
+ represents the concentration measured in the original literature without undergoing dry-to-wet weight conversion;
Abbreviation: HILIC-UPLC: Ultra Performance Liquid Chromatography-Hydrophilic Interaction Chromatography; LC-MS: Liquid Chromatography Mass Spectrometry; LC-MS/MS: Liquid Chromatography-Tandem Mass Spectrometry.
