# Supplementary material for: Dietary Exposure and Risk Assessment for L-Ergothioneine in China
Source: Foods. 2026 Mar 1;15(5):822. doi: 10.3390/foods15050822 (PMC12985124; doi:10.3390/foods15050822)
Supplement: Supplementary file 1 [file foods-15-00822-s001.zip › Table S2.pdf]

Table S2. Dietary intake levels of L-EGT in different regions (mg/kg bw/d).

Province/Autonomous Region/Municipality	General population (n=42218)			Consuming population (n=10518)		
	n	mean	P_{95}	n	mean	P_{95}
Shanghai	369	0.063	0.192	196	0.118	0.489
Yunnan	1,881	0.054	0.125	386	0.265	1.038
Inner Mongolia	2,054	0.032	0.027	282	0.236	0.622
Beijing	1,154	0.067	0.171	402	0.193	0.603
Jilin	344	0.030	0.087	110	0.095	0.321
Tianjin	693	0.055	0.154	270	0.140	0.473
Shandong	2,998	0.044	0.099	712	0.187	0.568
Guangdong	1,552	0.035	0.068	385	0.141	0.602
Guangxi	707	0.029	0.038	181	0.114	0.500
Jiangsu	3,253	0.037	0.095	854	0.140	0.545
Jiangxi	2,471	0.068	0.171	593	0.283	0.967
Hebei	2,115	0.020	0.021	353	0.120	0.446
Henan	2,551	0.034	0.090	522	0.167	0.488
Zhejiang	2,221	0.055	0.116	658	0.186	0.681
Hubei	3,689	0.043	0.143	801	0.198	0.678
Hunan	384	0.084	0.247	183	0.176	0.758
Gansu	1,825	0.032	0.058	383	0.150	0.593
Fujian	2,093	0.076	0.245	934	0.169	0.676
Guizhou	1,726	0.054	0.165	474	0.197	0.630
Liaoning	1,685	0.026	0.060	368	0.119	0.413
Chongqing	1,895	0.057	0.133	371	0.289	1.079
Shaanxi	2,291	0.028	0.088	607	0.106	0.358
Heilongjiang	2,267	0.020	0.037	493	0.094	0.323
