# Supplementary figures and images for: Dietary Exposure and Risk Assessment for L-Ergothioneine in China
Source: Foods. 2026 Mar 1;15(5):822. doi: 10.3390/foods15050822 (PMC12985124; doi:10.3390/foods15050822)

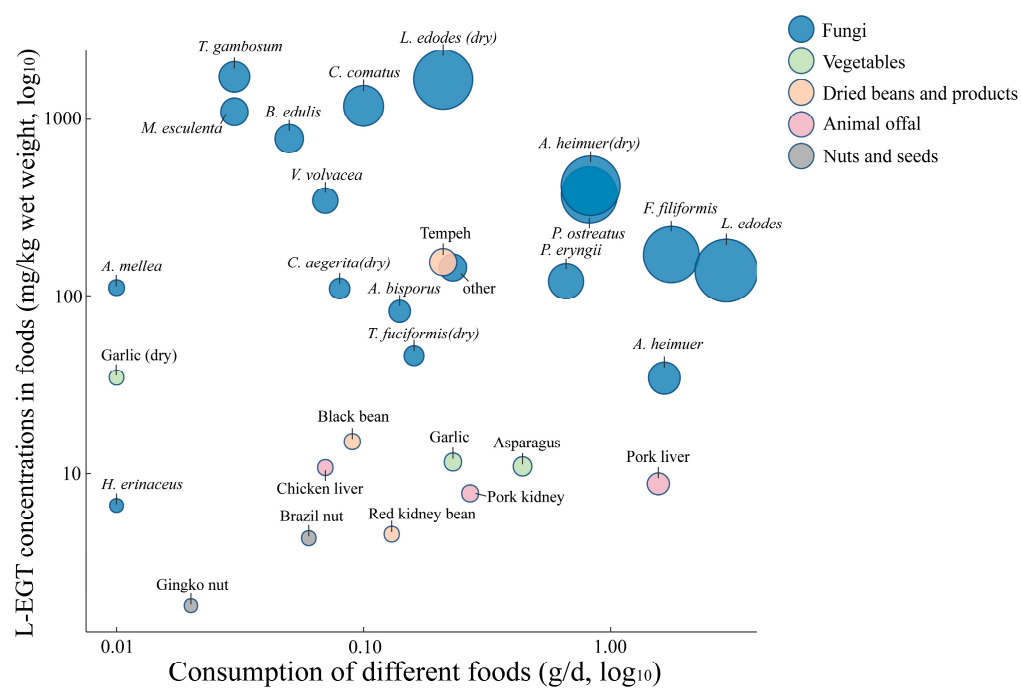

**Figure S1.** Bubble plot of the mean concentration of L-EGT in foods versus their consumption.

Supplement: Supplementary file 1 [file foods-15-00822-s001.zip › Figure S1.pdf]
